# Neck circumference as a predictor of metabolic syndrome, insulin resistance and low-grade systemic inflammation in children: the ACFIES study

**DOI:** 10.1186/s12887-016-0566-1

**Published:** 2016-03-08

**Authors:** Diego Gomez-Arbelaez, Paul Anthony Camacho, Daniel Dylan Cohen, Sandra Saavedra-Cortes, Cristina Lopez-Lopez, Patricio Lopez-Jaramillo

**Affiliations:** Dirección de Investigaciones, Fundación Oftalmológica de Santander - FOSCAL, Floridablanca, Colombia; Instituto MASIRA, Facultad de la Ciencias de la Salud, Universidad de Santander - UDES, Bucaramanga, Colombia; Departamento de Endocrinología, Escuela de Medicina, Universidad de Santiago de Compostela, Santiago de Compostela, España; Escuela de Medicina, Universidad Autónoma de Bucaramanga – UNAB, Bucaramanga, Colombia; Fundación Oftalmológica de Santander - FOSCAL, Calle 155A N. 23–09, El Bosque, Floridablanca, Santander Colombia

**Keywords:** Childhood obesity, Anthropometric measurements, Neck circumference, Metabolic syndrome, Low-grade systemic inflammation, Insulin resistance, Cardiometabolic risk, Latin America, Colombia

## Abstract

**Background:**

The current study aims to evaluate the association between neck circumference (NC) and several cardio-metabolic risk factors, to compare it with well-established anthropometric indices, and to determine the cut-off point value of NC for predicting children at increased risk of metabolic syndrome, insulin resistance and low-grade systemic inflammation.

**Methods:**

A total of 669 school children, aged 8–14, were recruited. Demographic, clinical, anthropometric and biochemical data from all patients were collected. Correlations between cardio-metabolic risk factors and NC and other anthropometric variables were evaluated using the Spearman’s correlation coefficient. Multiple linear regression analysis was applied to further examine these associations. We then determined by receiver operating characteristic (ROC) analyses the optimal cut-off for NC for identifying children with elevated cardio-metabolic risk.

**Results:**

NC was positively associated with fasting plasma glucose and triglycerides (*p* = 0.001 for all), and systolic and diastolic blood pressure, C-reactive protein, insulin and HOMA-IR (*p* < 0.001 for all), and negatively with HDL-C (*p* = 0.001). Whereas, other anthropometric indices were associated with fewer risk factors.

**Conclusions:**

NC could be used as clinically relevant and easy to implement indicator of cardio-metabolic risk in children.

## Background

The prevalence of obesity in children and adolescents is increasing worldwide and it is now recognized as an international public health concern [1]. Epidemiological and clinical investigations have revealed that the association between obesity and cardiovascular and metabolic risk factors begins early in life [2, 3]. Childhood obesity is associated with increased prevalence of hypertension, dyslipidemia, and abnormal glucose tolerance [2–4]. Thus, identifying and controlling childhood obesity is an important goal in the prevention of cardiovascular diseases (CVD) in later life [5].

Although obesity is at the core of the development of CVD, appropriate anthropometric measures and cut-off points to identify children with elevated cardio-metabolic risk factors are not well established. The most widely used method to categorize overweight and obese children and to predict cardiovascular and metabolic risk is the body mass index (BMI) [6]. However, BMI has been considered as an imperfect measure of adiposity, because it does not distinguish between muscle mass and fat mass, and requires calculations and the use of charts that may not always be available [7, 8].

Alternative measures to BMI such as waist-to-hip ratio (WHR) and waist circumference, which also give some indication of fat distribution, have been used as alternatives, but none of these have been accepted as a gold standard measure to identify cardiovascular and metabolic risk [9, 10]. Both have limitations in distinguishing the contribution from ectopic adipose tissue and subcutaneous adipose tissue [11], which show strong and modest correlations to cardio-metabolic risk, respectively [12, 13].

Prior studies have suggested that upper body fat plays a role in cardio-metabolic risk [14, 15], and neck circumference (NC) was proposed as a new measurement to evaluate overweight and obesity in children [16–18]. NC has demonstrated to be an independent predictor of metabolic risk beyond BMI and waist circumference [15] and to be positively associated with insulin resistance and visceral adipose tissue in adults [19], but few studies have been conducted to determine its association with cardio-metabolic risk factors in children [20, 21]. Hence, the aims of the present study were to evaluate the association between NC and several cardio-metabolic risk factors and to compare these associations with those of BMI and other well-established anthropometric indexes in a Latin American pediatric population.

## Methods

### Study population

During the 2011–2012 school year, we conducted the cross-sectional component of the ACFIES study (Association between Cardiorespiratory Fitness, Muscular Strength and Body Composition with Metabolic Risk Factors in Colombian Children) to identify the prevalence and associations of cardiovascular risk factors, in a sample of schoolchildren from both sexes, enrolled in public elementary and high schools (grades 5 and 6), from the city of Bucaramanga, Colombia. All the recruited participants met the general ACFIES inclusion criteria: age range 8 to 14 years, not having any physical disability and be free of any acute infection lasting less than 2 weeks before the inclusion. Moreover, children were excluded if were using medications that could alter blood pressure, insulin resistance, glycemic levels and/or lipid profile. The study protocol was in accordance with the Declaration of Helsinki and was approved by the Health Research Ethics Board of the Ophthalmological Foundation of Santander (FOSCAL). The children expressed their interest in participating in the study, and parents or legal guardians gave written informed consent, before the children were included in the study.

### Anthropometric measurements and physical examination

All physical assessments and anthropometric measurements were performed after an overnight fast (8 to 10 h), in duplicate by well-trained health workers. For the analysis we used the mean of the two measurements. Participant’s body weight was measured to the nearest 0.1 kg on an electronic device (Tanita BC544, Tokyo, Japan), in underwear and without shoes, and height was measured to the nearest 0.1 cm using a mechanical stadiometer with platform (Seca 274, Hamburg, Germany), while participants were asked to stand erect with their head positioned in the Frankfort horizontal plane. BMI was calculated by dividing body weight by the square of height (BMI = weight (kg)/height (m)^2^). The weight status was classified according to Barlow et al. [22].

Neck circumference was measured to the nearest 0.1 cm using a tape measure. The superior border of the tape measure was placed just below the laryngeal prominence and applied perpendicular to long axis of the neck. Waist circumference was determined at the middle point between the lower edge of the ribs and the iliac anterior spine. The measurement was made at the end of a normal expiration while the subject stood upright. Hip circumference was measured over non-restrictive underwear at the level of the maximum extension of the buttocks posteriorly in a horizontal plane. All circumferences were measured using a measuring tape with spring scale (Ohaus 8004-MA, NJ, USA). WHR was calculated as waist circumference divided by hip circumference. Waist-to-height ratio (WHtR) was calculated by dividing waist circumference by height in cm. The measurements were realized according to the procedures previously described by Lohman et al. [23].

Skinfold thickness was measured to the nearest 0.2 mm on the right side of the body at the triceps and subscapular sites using a skinfold caliper (Harpenden C-136, United Kingdom) and body fat percentage (%BF-Skinfold) estimated using skinfold equations described by Slaughter et al [24]. Body fat percentage was also assessed by bioelectrical impedance analysis (BIA) (%BF-BIA) (Tanita BC544, Tokyo, Japan). Systolic blood pressure and diastolic blood pressure were determined after a resting period of 10 min in the sitting position using an automatic and calibrated sphygmomanometer with a pediatric cuff (Omron HEM 757 CAN, Hoofddorp, Netherlands). Pubertal development was assessed by Tanner stage of breast development in girls and testicular volume in boys [25].

### Biochemical parameters

Venous blood samples were collected in the morning at the same time (07:00 am to 09:00 am), after an overnight fast (8 to 10 h), and from the antecubital vein. Participants were asked not to do any prolonged exercise during the 24 h prior to the exam. Blood samples were analyzed for concentrations of fasting plasma glucose and lipid profile (total cholesterol, triglycerides, and high-density lipoprotein cholesterol (HDL-C)) using a routine colorimetric method (Biosystems BTS-303 Photometric, Barcelona, Spain). High-sensitivity C-reactive protein (hs-CRP) was quantified using a turbid metric test (SPINREACT, Spain), and insulin levels were determined using an insulin microplate ELISA test (Monobind, USA). Samples were processed and analyzed in the clinical laboratory of bacteriology school of the University of Santander - UDES.

Homeostasis model assessment for insulin resistance (HOMA-IR) was calculated using the equation: HOMA-IR = Fasting insulin (lU/ml) x Fasting glucose (mg/dl)/405 [26].

### Cardiovascular and metabolic risk definition

For this study, the cardiovascular and metabolic risk in children and adolescents was defined according to a modified version of the National Health and Nutrition Examination Survey (NHANES) definition of metabolic syndrome (MetS) [27]. The considered parameters were: increased waist circumference (≥75th percentile for age and sex of study cohort), elevated triglycerides (≥110 mg/dl), low HDL-C (≤40 mg/dl), elevated systolic blood pressure and/or diastolic blood pressure (≥90 percentile for age, sex and height), and elevated fasting plasma glucose (≥100 mg/dl). MetS was defined by the presence of 3 or more of the above criteria [27]. Although the NHANES definition was not intended to be applied to children below 12 years of age, for the purposes of this study to enable comparisons to be made and as cardiovascular and metabolic alterations can be present in children from their earliest years of life [2, 3], we have defined the individual risk components of MetS across the complete sample of children aged between 8 to 14 years. Moreover, a value of ≥2.6 in HOMA-IR was considered to indicate insulin resistance [28], and values of hs-CRP ≥0.55 mg/dl (75^th^ percentile in our study sample) were considered as low-grade systemic inflammation.

### Statistical analysis

Descriptive statistics were computed for variables of interest, and included mean values and standard deviations of continuous variables and absolute and relative frequencies of categorical factors. Normality of distribution was checked for continuous variables using the Shapiro-Wilk test and by graphical methods. Student’s *t*-test and Mann-Whitney test were used to assess potential differences in continuous variables. We tested for differences in categorical variables using the Pearson’s chi-squared test (Chi^2^). Correlations between cardio-metabolic risk factors and anthropometric variables were evaluated using the Pearson’s correlation or Spearman’s correlation coefficient, according to normality of distributions. Multiple linear regression analysis was applied to further examine these associations.

For selection of the cut-off points of NC that could identify MetS, insulin resistance and low-grade systemic inflammation according to gender, analyzes were made using the ROC (receiver operating characteristic) curves. The statistical significance of each analysis was verified by the area under the ROC curve (AUCs) and by 95 % confidence intervals (95 % CI´s). The maximum values of the Youden’s index [29] were used as a criterion for selecting the optimum cut-off points. All statistical analyzes were carried out using Stata statistical software, release 11.0 (Stata Corporation, College Station, TX, USA). A *p* < 0.05 was considered statistically significant.

## Results

### Descriptive statistics

**Fig. 1:**
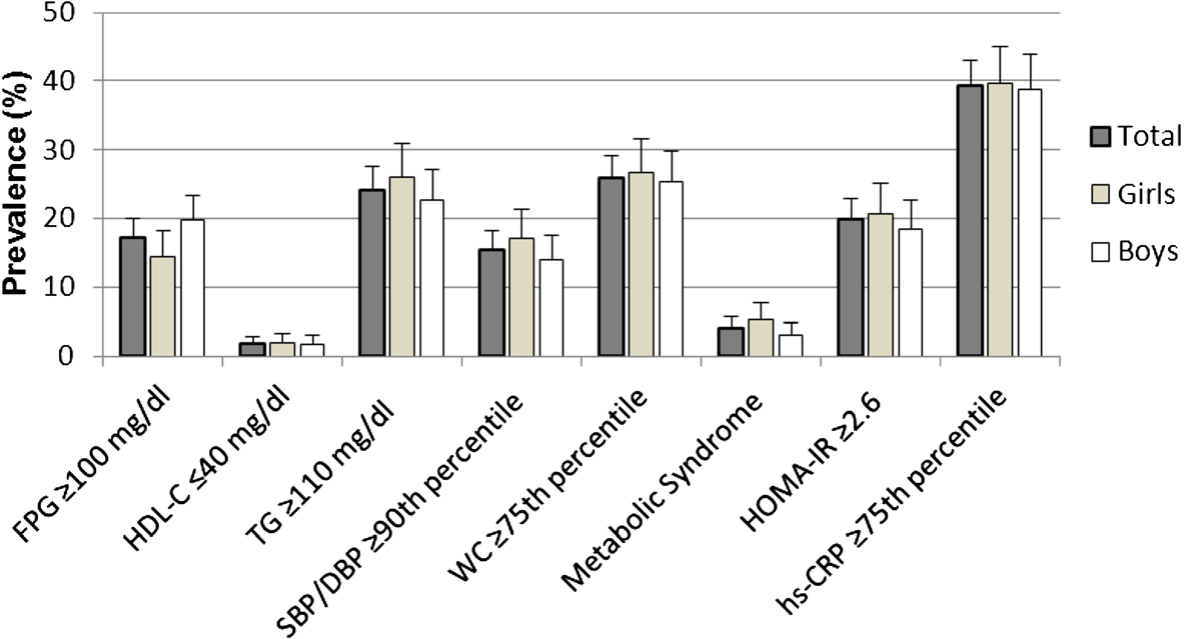
Prevalence of metabolic syndrome and its components, insulin resistance and low-grade systemic inflammation among study population. Data are presented as relative frequencies with 95 % confidence intervals represented by vertical bars. Significant differences between girls and boys (Pearson’s chi-squared test (Chi^2^)). FPG: fasting plasma glucose; HDL-C: high-density lipoprotein cholesterol; TG: triglycerides; SBP: systolic blood pressure; DBP: diastolic blood pressure; WC: waist circumference; hs-CRP: high sensitivity C-reactive protein

As it has been previously reported [30, 31], a total of 669 children and adolescents were recruited during the cross-sectional component of the ACFIES study, of which 351 (52.5 %) were boys. The overall mean age was 11.5 ± 1.1 years. Demographic, anthropometric and metabolic characteristics of the study population by sex are presented in Table 1. Compared to the girls, mean systolic blood pressure, waist circumference, WHR, WHtR, NC and %BF-Skinfold were significantly higher, while height, %BF-BIA, triglycerides, insulin and HOMA-IR were significantly lower in boys. Among our study population, 85 (12.9 %) were overweight and 65 (9.8 %) were obese. There were no statistically significant differences in weight status and BMI between both genders. Sex-specific prevalences of MetS and its individual abnormalities, insulin resistance and low-grade systemic inflammation were also estimated (Fig. 1), and statistical differences were not found.

**Table 1:** Demographic, anthropometric and metabolic data

	Total (*n* = 669)	Girls (*n* = 318)	Boys (*n* = 351)
Age (years)^a^	11.52 ± 1.13	11.52 ± 1.10	11.51 ± 1.16
SBP (mmHg)^a^	114.51 ± 11.59	113.29 ± 11.72	115.58 ± 11.38^b^
DBP (mmHg)^a^	73.78 ± 9.47	73.66 ± 8.97	73.86 ± 9.93
Anthropometric measures^a^			
Weight (kg)	40.08 ± 10.07	40.33 ± 9.77	39.86 ± 10.35
Height (m)	1.45 ± 0.09	1.45 ± 0.08	1.44 ± 0.09^b^
BMI (kg/m^2^)	18.87 ± 3.61	18.81 ± 3.52	18.93 ± 3.68
WC (cm)	65.95 ± 9.73	64.86 ± 9.02	66.92 ± 10.24^b^
WHR	0.84 ± 0.08	0.81 ± 0.06	0.86 ± 0.09^b^
WHtR	0.45 ± 0.06	0.44 ± 0.06	0.46 ± 0.06^b^
NC (cm)	29.93 ± 2.39	28.40 ± 2.06	29.41 ± 2.55^b^
%BF-BIA	20.47 ± 7.50	22.71 ± 6.89	18.43 ± 7.46^b^
%BF-Skinfold	25.47 ± 11.37	24.63 ± 9.10	26.23 ± 13.04^b^
Z-score BMI (kg/m^2^)	-0.0004 ± 0.98	-0.0008 ± 0.98	-5.45^-7^ ± 0.98
Z-score WC (cm)	-0.038 ± 0.99	4.88^-8^ ± 0.99	-0.073 ± 0.99^b^
Biochemical measurements^a^			
FPG (mg/dl)	88.52 ± 12.56	87.87 ± 12.32	89.12 ± 12.76
TC (mg/dl)	159.23 ± 39.28	158.25 ± 39.04	160.13 ± 39.53
HDL-C (mg/dl)	75.34 ± 19.96	74.57 ± 19.82	76.04 ± 20.08
TG (mg/dl)	91.76 ± 52.37	94.07 ± 46.79	89.67 ± 56.97^b^
hs-CRP (mg/dl)	0.89 ± 1.62	0.88 ± 1.52	0.89 ± 1.71
Insulin (lU/ml)	2.58 ± 2.61	2.91 ± 2.91	2.29 ± 2.26^b^
HOMA-IR	0.57 ± 0.58	0.64 ± 0.66	0.50 ± 0.50^b^
Weight status (*n* - %)^d^			
o Underweight	29 (4.4)	9 (2.9)	20 (5.8)
o Normal weight	479 (72.8)	240 (77.2)	239 (68.9)
o Overweight	85 (12.9)	42 (13.5)	43 (12.4)
o Obese	65 (9.8)	20 (6.4)	45 (12.9)
Tanner stage (*n* - %)^e^			
o 1	368 (56.3)	149 (47.8)	219 (64.0)^c^
o 2	208 (31.8)	110 (35.3)	98 (28.7)
o 3	78 (11.9)	53 (16.9)	25 (7.3)

*SBP* systolic blood pressure, *DBP* diastolic blood pressure, *BMI* body mass index, *WC* waist circumference, *WHR* waist-to-hip ratio, *WHtR* waist-to-height ratio, *NC* neck circumference, *%BF-BIA* body fat percentage – bioelectrical impedance analysis, *%BF-Skinfold* body fat percentage – skinfolds, *FPG* fasting plasma glucose, *TC* total cholesterol, *HDL-C* high-density lipoprotein cholesterol, *TG* triglycerides, *hs-CRP* high sensitivity C-reactive protein

^a^Data are presented as mean ± standard deviation for continuous variables. ^b^Mann-Whitney test *p* < 0.05. ^c^Pearson’s chi-squared test (Chi^2^) *p* <0.05

^d^data missing for 11 participants

^e^data missing for 15 participants

### Correlation between anthropometric indexes and cardio-metabolic risk factors

Correlations of anthropometric indexes and cardio-metabolic risk factors are presented in Table 2 for the total sample and by gender. Z-score BMI was positively correlated with triglycerides, systolic and diastolic blood pressure, hs-CRP, insulin and HOMA-IR in both genders, and inversely correlated with HDL-C only in boys. Z-score WC was positively correlated with triglycerides, systolic and diastolic blood pressure, insulin and HOMA-IR in both genders, with fasting plasma glucose and hs-CRP only in girls, and inversely correlated with HDL-C only in boys. WHR was positively correlated only with triglycerides in both genders, with diastolic blood pressure, insulin and HOMA-IR only in boys, and with hs-CRP only in girls. WHtR was positively correlated with triglycerides, systolic and diastolic blood pressure, insulin and HOMA-IR in both genders, and with hs-CRP only in girls. %BF-BIA was positively correlated with triglycerides, systolic and diastolic blood pressure, insulin and HOMA-IR in both genders, with hs-CRP only in girls, and inversely correlated with HDL-C only in girls. %BF-Skinfold was positively correlated with systolic and diastolic blood pressure, hs-CRP, insulin and HOMA-IR in both genders, with triglycerides only in boys, and inversely correlated with HDL-C in both genders. NC was positively correlated with fasting plasma glucose, systolic and diastolic blood pressure, hs-CRP, insulin and HOMA-IR in both genders, with triglycerides only in boys, and inversely correlated with HDL-C in both genders.

**Table 2:** Correlations between cardiometabolic risk factors and anthropometric measurements according to gender

	Z-score BMI			Z-score WC			Waist to hip ratio			Waist to height ratio		
	Total	Girls	Boys	Total	Girls	Boys	Total	Girls	Boys	Total	Girls	Boys
FPG (mg/dl)	0.08	0.10	0.06	0.10*	0.13*	0.08	-0.04	-0.08	-0.07	0.02	0.01	0.02
HDL-C (mg/dl)	-0.12*	-0.09	-0.15*	-0.16**	-0.10	-0.21**	0.02	0.01	-0.02	-0.06	-0.08	-0.07
TG (mg/dl)	0.16**	0.15*	0.17*	0.23**	0.19*	0.25**	0.10*	0.12*	0.20**	0.15**	0.15*	0.19**
SBP (mmHg)	0.29**	0.28**	0.30**	0.31**	0.31**	0.33**	0.08*	0.01	0.04	0.21**	0.17*	0.21**
DBP (mmHg)	0.35**	0.36**	0.33**	0.35**	0.33**	0.37**	0.06	-0.03	0.17*	0.29**	0.26**	0.33**
hs-CRP (mg/dl)	0.15*	0.19*	0.12*	0.15**	0.21**	0.09	0.04	0.12*	0.01	0.14**	0.22**	0.08
Insulin (Ul/ml)	0.27**	0.28**	0.27**	0.29**	0.23**	0.33**	0.06	0.06	0.23**	0.24**	0.21**	0.34**
HOMA-IR	0.27**	0.27**	0.26**	0.29**	0.24**	0.33**	0.05	0.03	0.21**	0.24**	0.20*	0.33**
	%BF – BIA			%BF - Skinfold			Neck Circumference					
	Total	Girls	Boys	Total	Girls	Boys	Total	Girls	Boys			
FPG (mg/dl)	0.01	0.09	-0.03	0.06	0.10	0.04	0.20**	0.19**	0.19**			
HDL-C (mg/dl)	-0.09*	-0.12*	-0.02	-0.12*	-0.11*	-0.13*	-0.19**	-0.12*	-0.29**			
TG (mg/dl)	0.18**	0.13*	0.17*	0.15**	0.09	0.19**	0.11*	0.10	0.18*			
SBP (mg/dl)	0.17**	0.26**	0.20**	0.24**	0.25**	0.23**	0.39**	0.34**	0.42**			
DBP (mg/dl)	0.33**	0.39**	0.32**	0.35**	0.41**	0.30**	0.29**	0.29**	0.31**			
hs-CRP (mg/dl)	0.11*	0.18*	0.05	0.16**	0.18*	0.15*	0.15**	0.17*	0.15*			
Insulin (Ul/ml)	0.30**	0.24**	0.29**	0.28**	0.21**	0.33**	0.19**	0.22**	0.25**			
HOMA-IR	0.28**	0.24**	0.27**	0.28**	0.23**	0.32**	0.21**	0.23**	0.27**			

*Spearman’s correlation coefficient *p* < 0.05. **Spearman’s correlation coefficient *p* < 0.001

*BMI* body mass index, *WC* waist circumference, *%BF-BIA* body fat percentage – bioelectrical impedance analysis, *%BF-Skinfold* body fat percentage – skinfolds, *FPG* fasting plasma glucose, *HDL-C* high-density lipoprotein cholesterol, *TG* triglycerides, *SBP* systolic blood pressure, *DBP* diastolic blood pressure, *hs-CRP* high sensitivity C-reactive protein

### Multiple linear regression analysis between anthropometric indexes and cardio-metabolic risk factors

Table 3 illustrates the results of the multivariate regression analysis conducted using separately each CVD risk factor as the dependent variable and controlling for age, gender and Tanner stage. Fating plasma glucose was significantly associated only with NC, and HDL-C was associated with waist circumference and NC. In contrast, triglycerides, hs-CRP, insulin and HOMA-IR were significantly associated with all the anthropometric indices, whereas systolic and diastolic blood pressures were associated with all the anthropometric indices, except WHR.

**Table 3:** Multiple linear regression analysis, using each cardiometabolic risk factor as the dependent variable

Dependent variable	Independent factor	Coef. ± SE	*P* Value	Dependent variable	Independent factor	Coef. ± SE	*P* Value
FPG (mg/dl)	BMI (kg/m^2^)	0.193 ± 0.149	0.194	DBP (mmHg)	BMI (kg/m^2^)	0.849 ± 0.106	<0.001
	WC (cm)	0.100 ± 0.056	0.075		WC (cm)	0.346 ± 0.039	<0.001
	WHR	-12.777 ± 6.791	0.060		WHR	7.498 ± 5.024	0.136
	WHtR	2.198 ± 8.620	0.799		WHtR	46.847 ± 6.094	<0.001
	%BF-BIA	0.063 ± 0.075	0.399		%BF-BIA	0.500 ± 0.051	<0.001
	%BF-Skinfold	0.039 ± 0.045	0.393		%BF-Skinfold	0.283 ± 0.031	<0.001
	NC (cm)	0.815 ± 0.244	0.001		NC (cm)	1.305 ± 0.173	<0.001
HDL-C (mg/dl)	BMI (kg/m^2^)	-0.279 ± 0.236	0.237	hs-CRP (mg/dl)	BMI (kg/m^2^)	0.132 ± 0.020	<0.001
	WC (cm)	-0.237 ± 0.088	0.008		WC (cm)	0.043 ± 0.007	<0.001
	WHR	-6.135 ± 10.745	0.568		WHR	2.459 ± 0.936	0.009
	WHtR	-21.145 ± 13.584	0.120		WHtR	7.225 ± 1.177	<0.001
	%BF-BIA	-0.149 ± 0.116	0.203		%BF-BIA	0.061 ± 0.010	<0.001
	%BF-Skinfold	-0.133 ± 0.072	0.067		%BF-Skinfold	0.037 ± 0.006	<0.001
	NC (cm)	-1.333 ± 0.384	0.001		NC (cm)	0.133 ± 0.034	<0.001
TG (mg/dl)	BMI (kg/m^2^)	2.149 ± 0.621	0.001	Insulin (Ul/ml)	BMI (kg/m^2^)	0.245 ± 0.031	<0.001
	WC (cm)	1.253 ± 0.229	<0.001		WC (cm)	0.108 ± 0.011	<0.001
	WHR	104.268 ± 28.023	<0.001		WHR	5.850 ± 1.448	<0.001
	WHtR	155.901 ± 35.351	<0.001		WHtR	14.963 ± 1.785	<0.001
	%BF-BIA	1.260 ± 0.313	<0.001		%BF-BIA	0.130 ± 0.015	<0.001
	%BF-Skinfold	0.754 ± 0.190	<0.001		%BF-Skinfold	0.086 ± 0.009	<0.001
	NC (cm)	3.887 ± 1.014	<0.001		NC (cm)	0.362 ± 0.051	<0.001
SBP (mmHg)	BMI (kg/m^2^)	0.705 ± 0.129	<0.001	HOMA-IR	BMI (kg/m^2^)	0.055 ± 0.007	<0.001
	WC (cm)	0.311 ± 0.048	<0.001		WC (cm)	0.024 ± 0.002	<0.001
	WHR	7.001 ± 6.020	0.245		WHR	1.192 ± 0.330	<0.001
	WHtR	34.576 ± 7.503	<0.001		WHtR	3.306 ± 0.407	<0.001
	%BF-BIA	0.362 ± 0.064	<0.001		%BF-BIA	0.029 ± 0.003	<0.001
	%BF-Skinfold	0.206 ± 0.039	<0.001		%BF-Skinfold	0.018 ± 0.002	<0.001
	NC (cm)	1.719 ± 0.205	<0.001		NC (cm)	0.085 ± 0.011	<0.001

After controlling for age, gender and Tanner stage

*FPG* fasting plasma glucose, *HDL-C* high-density lipoprotein cholesterol, *TG* triglycerides, *SBP* systolic blood pressure, *DBP* diastolic blood pressure, *hs-CRP* high sensitivity C-reactive protein, *BMI* body mass index, *WC* waist circumference, *WHR* waist-to-hip ratio, *WHtR* waist-to-height ratio, *%BF-BIA* body fat percentage – bioelectrical impedance analysis, *%BF-Skinfold* body fat percentage – skinfolds, *NC* neck circumference

### Neck circumference cut-off points to identify MetS, insulin resistance and low-grade systemic inflammation according to gender

The cut-off points and respective sensitivity and specificity values, the AUCs and the Youden’s index of NC for the identification of MetS, insulin resistance and low-grade systemic inflammation according to gender are shown in Table 4. NC cut-off values for MetS were calculated to be 28.5 cm (95 % CI, 0.68 – 0.78) in girls and 29 cm (95 % CI, 0.68 – 0.78) in boys, 29.3 cm (95 % CI, 0.49 – 0.60) in girls and 29.2 (95 % CI, 0.47 – 0.58) in boys for detecting low-grade systemic inflammation, and 29 cm (95 % CI, 0.51 – 0.62) in girls and 30 cm (95 % CI, 0.49 – 0.59) in boys for identifying insulin resistance (Table 5).

**Table 4:** Neck circumference cut-offs points to identify metabolic syndrome, low-grade systemic inflammation and insulin resistance in study sample according to gender

	Cutoffs (cm)	Sensitivity (%)	Specificity (%)	AUC (IC 95 %)	Youden’s index
Metabolic Syndrome					
Girls	28.5	87.50	53.61	0.73 (0.68 - 0.78)	0.41
Boys	29	100	45.37	0.74 (0.68 - 0.78)	0.45
Low-grade systemic inflammation					
Girls	29.3	42.02	69.15	0.55 (0.49 - 0.60)	0.11
Boys	29.2	54.62	51.63	0.53 (0.47 - 0.58)	0.06
Insulin resistance					
Girls	29	50.00	62.35	0.57 (0.51 - 0.62)	0.12
Boys	30	52.54	61.19	0.54 (0.49 - 0.59)	0.13

Receiver operating characteristic (ROC) analyzes. Youden’s index = Sensitivity + Specificity – 1

**Table 5:** Advantages and limitations in pediatric population of anthropometrics measurements to identify metabolic alterations

	FPG	HDL-C	TG	SPB/DPB	hs-CRP	Insulin	HOMA-IR
BMI	-	+	++	++	++	++	++
WC	+	+	++	++	++	++	++
WHR	-	-	++	+	+	+	+
WHtR	-	-	++	++	+	++	++
%BF-BIA	-	+	++	++	+	++	++
%BF-Skinfold	-	++	+	++	++	++	++
NC	++	++	+	++	++	++	++

(-) Not correlation; (+) Correlation in girls or boys; (++) Correlation in both girls and boys

*FPG* fasting plasma glucose, *HDL-C* high-density lipoprotein cholesterol, *TG* triglycerides, *SBP* systolic blood pressure, *DBP* diastolic blood pressure, *hs-CRP* high sensitivity C-reactive protein, *BMI* body mass index, *WC* waist circumference, *WHR* waist-to-hip ratio, *WHtR* waist-to-height ratio, *%BF-BIA* body fat percentage – bioelectrical impedance analysis, *%BF-Skinfold* body fat percentage – skinfolds, *NC* neck circumference

## Discussion

We found that NC was associated with all the assessed cardio-metabolic risk factors similar to that observed for waist circumference, which was associated with all the cardio-metabolic risk factors except fasting plasma glucose. The association for HDL-C was more robust for NC than for waist circumference. The other anthropometric indices were not associated neither with fasting plasma glucose nor HDL-C, and WHR was also not associated with systolic and diastolic blood pressure. Interestingly, similar NC cut-off points for identifying children at elevated risk of MetS, insulin resistance and low-grade systemic inflammation were obtained by gender (28.5 to 29.3 cm in girls and 29 to 30 cm in boys), making it a simple marker of metabolic risk. Therefore, NC is a measure that potentially might be implemented in situations where equipment availability or cultural issues limit the use of the traditional anthropometric measures.

Moreover, it should be noted that in cases wherein significant associations were found, most of the anthropometric measures were similar to each other in the strength of these associations. Thus, our results confirm the value of a complete anthropometric assessment in the identification of cardiovascular and metabolic risk factors in children.

Adiposity is widely accepted to play a key role in the pathogenesis of cardiovascular and metabolic diseases in children [3–5, 32]. So, it is important the identification of overweight children with cardio-metabolic risk factors in whom counseling and treatment must be provided in a timely manner. The determination of biochemical variables is costly, making impractical its use as a screening tool, particularly in low-middle income countries with lower resources. Thus, the present findings showing that NC, which only requires a tape measure, is effective, simple, easy-to-use and inexpensive anthropometric measurement to identify children and adolescents with cardio-metabolic risk constitute an important contribution from a public health perspective.

However, previous studies [20, 21] have assessed the association between NC and cardio-metabolic risk in children, our study has the strength of having the largest pediatric population sample to date. Moreover, the results showed for the first time, an association between high NC and abnormal values of fasting plasma glucose and low-grade systemic inflammation. These results support the proposal of an increased cardio-metabolic risk in our population at lower levels of adiposity [33–35].

Although NC is an emerging marker of cardio-metabolic risk in children, it has been demonstrated as a good predictor of cardiovascular disease in adults with different conditions such as MetS, obstructive sleep apnea and fatty liver disease [15, 19, 36–39].

BMI has been the accepted standard measure of overweight and obesity for children two years of age and older [40]. However, some studies have suggested that BMI is not a good indicator of cardio-metabolic risk [7, 8, 41]. In our current study BMI was associated with most of the cardio-metabolic risk factors assessed, confirming that despite its apparent limitations, in children BMI is non inferior to measures that assess body composition and differentiate fat and lean mass, such as BIA or skinfolds [42]. We found that associations between BIA and skinfolds and cardio-metabolic risk factors were similar to that of the anthropometric indices; but, in contrast to NC, neither of these measures was associated with fasting plasma glucose and HDL-C. Moreover, it is notable that despite identical statistical associations with cardio-metabolic risk of these two field measures of body composition, the mean values were lower for BIA in boys and girls and %BF-BIA was significantly higher in girls than boys, while the reverse was the case for %BF-Skinfolds. Therefore, it is not clear which of these two estimates of %BF is more accurate or whether it is appropriate to calculate them using predictive equations validated in different populations.

Fat distribution is also recognized as an important determinant of metabolic risk [43] and those anthropometric measures such as waist circumference, WHR and WHtR are good indicators of visceral adipose tissue and therefore good predictors of cardiovascular risk [44–46]. In the present study, all these anthropometric indexes showed acceptable correlations with the cardio-metabolic risk factors, although none were superior to NC. Hence, in agreement with previous studies, we can also suggest the use of waist circumference, WHR and WHtR as an optional adiposity indexes in relation to the cardiovascular and metabolic health risk.

Our study should be interpreted in light of its limitations. First, is a cross-sectional study; therefore, the association with cardiovascular and metabolic disease outcomes could not be established. Second, as pubertal growth and development is characterized by changes in metabolic traits that characterize the MetS [47], we suggest further studies with larger sample sizes, in which the cut-off points would be defined by pubertal development. Third, we defined the cardio-metabolic risk using a modified NHANES definition of MetS, which we considered as the most applicable in the clinical practice based on the simplicity of its diagnostic criteria, however it should be mentioned that the appropriate risk factor cut-offs for children remain controversial, and therefore further studies to define thresholds for abnormalities of the metabolic components should be conducted. Fourth, our study was specifically conducted in a pediatric Latin American population. It has been proposed that fetal programming associated to maternal undernutrition, which prevalence still is high in Latin America, could affect the body composition and the utility of different anthropometrics measurements [35]. Hence, we believe that additional studies should be performed testing whether the proposed cut-offs points for NC are truly applicable in other populations and regions of the world.

## Conclusions

We evaluated the association between several cardio-metabolic risk factors and NC, a novel marker of risk, and compared this with classic anthropometric measures and indexes such as BMI and WHR and with field measures of body composition. While all of the anthropometric measures and indexes we assessed showed some associations with cardio-metabolic risk factors, including insulin resistance and low-grade systemic inflammation, we found that NC was the most consistent and robust marker. Further longitudinal studies in representative populations are required to confirm these findings and to establish NC as a basic criterion in the diagnosis of cardio-metabolic risk factors.
